# Comparative study: nonsynonymous and synonymous substitution of SARS-CoV-2, SARS-CoV, and MERS-CoV genome

**DOI:** 10.5808/gi.20058

**Published:** 2021-06-30

**Authors:** Vipan Kumar Sohpal

**Affiliations:** Department of Chemical & Bio Engineering, Beant College of Engineering & Technology, Gurdaspur 143521, India

**Keywords:** divergence time, respiratory syndrome, synonymous and nonsynonymous change, transition/transversion ratio

## Abstract

The direction of evolution can estimate based on the variation among nonsynonymous to synonymous substitution. The simulative study investigated the nucleotide sequence of closely related strains of respiratory syndrome viruses, codon-by-codon with maximum likelihood analysis, z selection, and the divergence time. The simulated results, dN/dS > 1 signify that an entire substitution model tends towards the hypothesis's positive evolution. The effect of transition/transversion proportion, Z-test of selection, and the evolution associated with these respiratory syndromes, are also analyzed. Z-test of selection for neutral and positive evolution indicates lower to positive values of dN-dS (0.012, 0.019) due to multiple substitutions in a short span. Modified Nei-Gojobori (P) statistical technique results also favor multiple substitutions with the transition/transversion rate from 1 to 7. The divergence time analysis also supports the result of dN/dS and imparts substantiating proof of evolution. Results conclude that a positive evolution model, higher dN-dS, and transition/transversion ratio significantly analyzes the evolution trend of severe acute respiratory syndrome coronavirus 2, severe acute respiratory syndrome coronavirus, and Middle East respiratory syndrome coronavirus.

## Introduction

Coronaviruses (CoVs) generally disturb human beings' respiratory tract and other mammals that causes severe respiratory infections. A previous study reveals that two extremely pathogenic human CoVs, including severe acute respiratory syndrome coronavirus (SARS-CoV) and Middle East respiratory syndrome coronavirus (MERS-CoV), growing from viral family, have led to worldwide epidemics at different times [1,2]. According to the World Health Organization, as of 15 Jan 2021, CoV had 13.1 million diagnosed cases causing 1.98 million deaths throughout the world [3]. A comparative study indicates that the ORF8 protein of SARS-CoV attained from host genes during evolution [4]. Comprehensive genomic analysis of severe acute respiratory syndrome coronavirus 2 (SARS-CoV-2) strains and its closely related coronavirus strains shows that the ratio of nucleotide to amino acid substitutions of the spike gene is higher [5]. Lower R0 values of MERS-CoV full genome assist in tracking transmission and multiple mutations [6]. Similarly, the complete genomic sequence of the porcine hamagglutinating encephalalomylitis virus discloses the existence of a truncated group ns2 gene [7]. Turkey coronavirus that has divergence within spike protein and provided the evidence of recombination that can directly lead to new coronaviruses [8]. The analysis was performed on 93 complete genomes of SARS-CoV-2 from the GISAID (Global Initiative for Sharing All Influenza Data) database to explore the evolution and human-to-human transmissions of SARS-CoV-2 [9]. The recombination study proved that the newly discovered MERS-CoV had obtained their spike genes from a bat coronavirus, which provide significant proof that bats represent MERS-CoV evolutionary origins [10]. Simulated 219 camels and human MERS-CoV genome sequences available in GenBank for Phylogenetic analysis showed that clade B divided into B1 to B6 (each containing both human and camel strains) [11]. Viral spike glycoprotein, which recognizes a cell surface receptor, supports that the SARS-CoV-2 a recombinant of the bat coronavirus and an unknown origin coronavirus. Higher sequence homology found between SARS-CoV-2 to bat CoV RaTG13 suggested that the Chinese chrysanthemum bat is the origin of SARS-CoV2 in China [12,13]. The Vietnam B-CoV, Cuban, and Chinese strains have high nucleotide sequence similarity due to same cluster [14]. Experimental data show that SARS-CoV-2 originate from bat CoV RaTG13 under the positive selection hypothesis model having common ancestor [15]. The inequality between nonsynonymous to synonymous changes simulates using capsid protein of human herpes virus to understand the evolution [16]. MEGA software implements numerous statistical techniques for nucleotide substitution models and estimates evolutionary rates [17]. The nonsynonymous mutations of SARS-CoV-2 were isolated and evaluated for surface glycoprotein spike for amino acid alterations [18]. The protein sequence similarity of pangolin-hCoV and bat-hCoV with human coronavirus was higher than their nucleotide similarity, denoting the occurrence of more synonymous mutations in the genome [19]. It has been observed that, due to the structure of the genetic code, nonsynonymous transitions are recessive than transversions to leads radical changes in amino acids [20]. In this simulative study, four nucleotide substitution methods simulated for revealing dN/dS and improved method, assumes that the substitution rate is not equal.

The purpose of the present work is to estimate the synonymous and nonsynonymous substitution in the genome of MERS-CoV, SARS-CoV, and SARS-CoV-2 with an objective to (1) normalization dN-dS value using codon through HyPhy using maximum likelihood approach (2) Z-test of selection for estimation of positive or neutral evolution using different transition/transversion ratio (tr/tv) (3) estimate the effect of different substitution models on divergence time.

## Methods

### Methodology and benchmark data

Nucleotide-coding sequences of SARS-CoV-2, SARS-Co-V, and MERS-CoV evaluate using the synonymous to nonsynonymous nucleotide substitutions of evolution model. The execution and assessment of substitution models assess using different statistical and simulated models under discrete conditions. MEGA used to simulated sequences SARS-CoV-2, SARS-Co-V, and MERS-CoV viruses that were deriving from the National Centre for Biotechnology Information (NCBI). The genomic data for substitution analysis of SARS-CoV-2 (NC_045512.2), MERS-CoV (NC_019843.3), and SARS-CoV (FJ588686.1) were retrieved from the NCBI GenBank database and filtered using BLAST. A genomic database that contains codons of SARS-CoV-2, SARS-CoV, and MERS-CoV is assuming that an approximate statistical and nucleotide substitution method must not diverge from dN/dS ratio. Furthermore, dN/dS ratio, additional parameters were also set in the simulation, including the normalized p-value, tr/tv, and divergence time.

## Results

### Phylogenetic analysis

**Fig. 1. f1-gi-20058:**
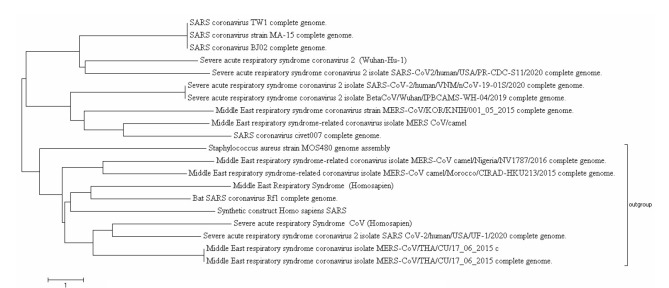
Phylogenetic tree using minimum-evolution method of severe acute respiratory syndrome coronavirus and Middle East respiratory syndrome coronavirus related genome family.

The 20 different strains of SARS-CoV and MERS-CoV are firstly assessed to detect the preliminary outgroup and similarity in genome sequence through MEGA software. The evolutionary history is inferred using the Minimum Evolution method as Fig. 1. The optimal tree with the sum of branch length is observed 60.85502865. The evolutionary distances were computed using the Maximum Composite Likelihood method and are in the units of the number of base substitutions per site. The minimum-evolution tree was searched using the Close-Neighbor-Interchange algorithm. The neighbor-joining algorithm is also used to generate the initial tree and analysis involved 20 nucleotide sequences. Codon positions included were 1st + 2nd + 3rd + Noncoding. All positions containing gaps and missing data were eliminated. There were a total of 1,692 positions in the final dataset.

The evolutionary tree inferences that severe acute respiratory syndrome coronavirus 2 (SARS-CoV-2) (Wuhan and USA strains) are extremely different severe acute respiratory syndrome coronavirus (SARS-CoV) and Middle East respiratory syndrome coronavirus (MERS-CoV) (Fig. 1). The genetic evolution can detect in divergent species. So, from the phylogenetic analysis its clear distinction between three selected strains SARS-CoV-2, SARS-CoV, and MERS-CoV for synonymous-to-synonymous substitution.

### dN/dS ratio and dN-dS

Selective strength, replicated in dN/dS, differs from biological sequence to sequence, nucleotide substitution model used for genomic analyses for the selection at codons level via HyPhy. GTR (General Time Reversible model), T-Nei (Tamura-Nei model), Fels1981 (Felsenstein 1981 model), and HKY (Hasegawa-Kishino-Yano model) models simulated to analyze the effects using sets of three biological sequences of respiratory syndrome virus. 1.136, 1.541, and 1.46 average values estimated for Fels1981, HKY, T-Nei, respectively. GTR model has the same value of dN/dS as estimates for the T-Nei. The average of normalized P, dN, and dS values for three respiratory syndrome sequences computed with the same four nucleotide substitution models and estimated values of dN and dS observed revealed in Table 1.

**Fig. 2.**
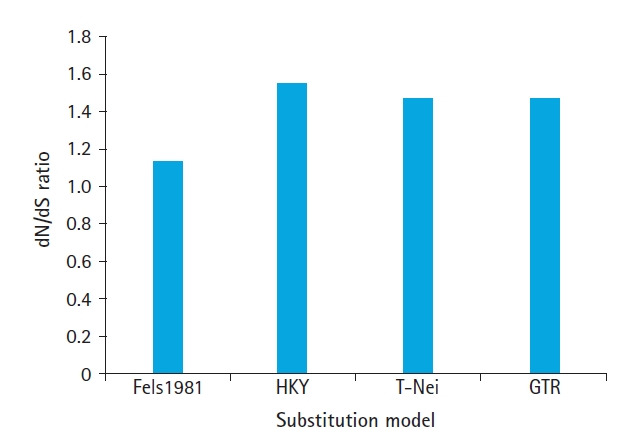
Estimated average dN/dS ratio under different substitution model for severe acute respiratory syndrome coronavirus 2, severe acute respiratory syndrome coronavirus, and Middle East respiratory syndrome coronavirus. Fels1981, Felsenstein 1981 model; HKY, Hasegawa-Kishino-Yano model; T-Nei, Tamura-Nei model; GTR, General Time Reversible model.

High dN/dS ratio observed for all nucleotide substitution models (Fels1981, HKY, T-Nei, GTR models), at codons level is larger than one tends towards to positive Darwinian selection. The values lie in the range from 1.136 to 1.541 as reproduced in Fig. 2. Similarly, higher dN-dS and positive values of statistic condition show an overabundance of nucleotide substitutions. The peak value dN-dS observe 737.25, and the lowest 227.38 corresponding to the HKY model and Fels1981. An intermediate value of dN-dS was found 694.43 for GTR and T-Nei model. A similar outcome obtains for normalized dN-dS, which assesses the expected substitutions per site. Results point out towards data sets of respiratory syndrome virus, normalized dN-dS, dN/dS ratio, and dN-dS for validating the positive selection hypothesis.

### Z-test of selection

Higher value of nonsynonymous to synonymous difference and ratio plays a significant role in the assessed positive evolution model. Z-test of selection is comparing the relative abundance of nonsynonymous to synonymous substitutions that occurred in the codon sequences. Codon-based analyses carry out for Z-test of selection for nucleotide sequences using the mathematical formula [17]:

$$Z=\frac{dN-dS}{\sqrt{Var(dS)\;+\;Var(dN)}}$$

Simulative outcomes are evaluated with PBL (Pamilo-Bianchi-Li method [K-2]), LWL (Li-Wu-Luo method [K-2]), NG (P) (Nei-Gojobori method [Proportion]), NG (JC) (Nei-Gojobori method [Juke Cantor]), and Kumar models for Z-test under constant tr/tv. Simulation performed of all the taxa of respiratory syndrome for dN > dS and dN = dS evolutionary hypothesis model. Table 2 explicates the average results of the respiratory syndrome for hypothesis sets. The outcome is approximately similar for both hypothesis models, but quantitative dN > dS giving confidence of positive evolution more dominant. The first two evolutionary models are based on p-distance and other three models are based on 0-fold, 2-fold, and 4-fold degenerate sites.

p-values NG (P) and Kumar method (K-2) are exceeded than 0.05 in the null hypothesis, but a comparatively lower p-value was found in the alternative hypothesis. p-values less than 0.05 considered a significant probability of decline in the null hypothesis in favor of the alternative positive model. The dN-dS parameter of both the hypothesized model except than NG (JC) model varies from 0.012 to 0.811 (dN = dS) and similarly bound between 0.019 to 0.831 dN > dS. The output parameter for the codon-based test of the respiratory syndrome is a positive evolution on the higher side, with the condition of dN > dS for most of the parameter combinations tested.

NG (P) and NG (JC) evolutionary models are widely used to measure evolution, based on the ratio of nonsynonymous distance (dN) to synonymous distance (dS), under the assumption that synonymous substitutions in the coding region are selectively neutral. In NG (P) methods, the main reason for this negative correlation is that there was a substantial proportion of codons with non-zero nonsynonymous difference but zero synonymous difference. The negative correlation appears to result mainly from three factors: (1) the occurrence of nucleotide differences varies stochastically across codons, (2) selection of purifying selection eliminating number nonsynonymous mutations, the rate of occurrence of nonsynonymous differences is much lower than that of synonymous differences, and (3) the overall rate of substitution is much less than one substitution per site. In the case of LWL (K-2), PBL (K 2), and Kumar method (K-2), 0-fold, 2-fold, and 4-fold degenerate codons, the proportion of codons with a non-zero nonsynonymous difference and the non-zero synonymous difference is higher.

### Effect of tr/tv

**Fig. 3.**
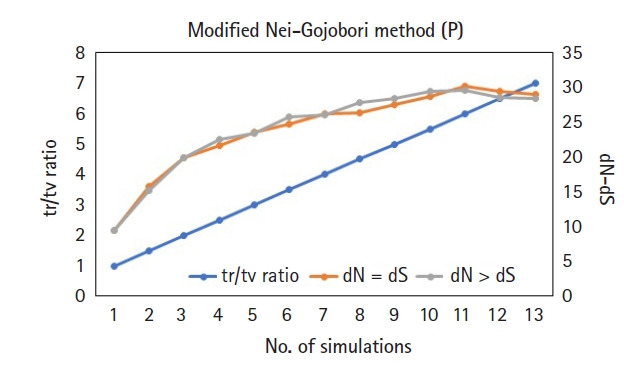
dN-dS vs. transition/transversion ratio (tr/tv) plot for number of simulations of severe acute respiratory syndrome coronavirus 2, severe acute respiratory syndrome coronavirus, and Middle East respiratory syndrome coronavirus.

The effects of the tr/tv with a set of three sequences of SARS-CoV-2, SARS-CoV, and MERS-CoV using MEGA were also investigated. The dN-dS distance calculates with the MNJ (P) method for two hypotheses of evolution plotted in Fig. 3. The tr/tv was assessed from 1.0 to 7.0. Positive evolution is less biased than neutral evolution, but both have approximately equivalent inclinations with an increasing tr/tv.

MNJ (P) gives the dN-dS value ranges from 9.5 to 30.21 and 9.397 to 29.66 for null and positive evolution models. The maximum dN-dS values observe for both methods, when tr/tv approach 6. With an increasing tr/tv (lower to higher), the results are changed moderately in both the evolutionary models. The tr/tv for genome restricted to 1 to 6, exceed than the upper limit no influence observes on dN-dS. We have made several observations to avoid divergence and to maintain consistency in evolution models. This is quite remarkable that neutral and positive evolution models show a comparatively similar performance.

### Effect of divergence time

**Fig. 4.**
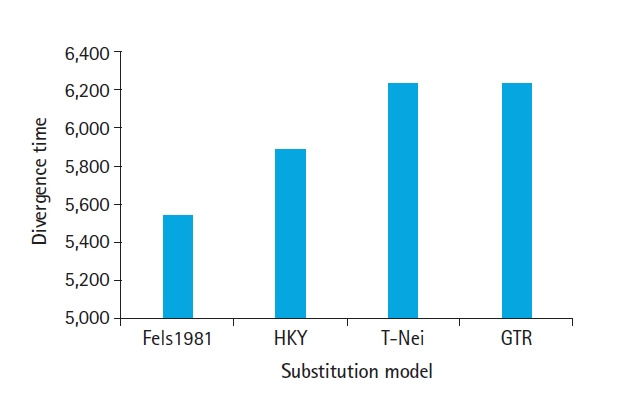
Estimated divergence time under different substitution model for severe acute respiratory syndrome coronavirus 2, severe acute respiratory syndrome coronavirus, and Middle East respiratory syndrome coronavirus. Fels1981, Felsenstein 1981 model; HKY, Hasegawa-Kishino-Yano model; T-Nei, Tamura-Nei model; GTR, General Time Reversible model. Fels1981, Felsenstein 1981 model; HKY, Hasegawa-Kishino-Yano model; T-Nei, Tamura-Nei model; GTR, General Time Reversible model.

The divergence time *t*=*f*(*dS,dN*) of nucleotide substitutions per codon. Three taxa of SARS-CoV-2, SARS-CoV, and MERS-CoV having an average of 1,181 codons that were used for simulation and tested four different models. The average estimates of divergence time against Fels 1981, HKY, T-Nei, and GTR are shown in Fig. 4. T-Nei and GTR models have nearly equal divergence times. Fels1981 is closely linked to the HKY model having a difference in divergence time of about 0.87% only, whereas the HKY model departs 5.58% from the T-Nei and GTR model in terms of divergence time value.

With increasing divergence time, dN-dS for positive evolution support by GTR and T-Nei, and biased approximation for neutral evolution model selection, while purifying evolution model remains unaffected by divergence. Figs. 2 and 4 compare the co-relation between dN/dS ratio raise and divergence time and directly proportional to each other. The outcomes of dN/dS indicate high divergence time, force towards the positive evolution model.

## Discussion

Numerous substitution models provide the platform for the comparative study of evolution for SARS-CoV-2, SARS-CoV, and MERS-CoV strains. The critical area is to analyze the synonymous and nonsynonymous substitution, divergence time, and the substitution ratio of nucleotide for the concept of evolution. Comparative analyses point out that overlook of substitution rate close to estimates of dN/dS parameters effect to evolutionary analysis. HKY model is robust in simulations and real datasets, especially concerning the Fels1981 model. The whole models have dN/dS > 1 expect diversity at the codon level is favored, to the positive evolution using the mutations. In Z-test selection of codon observed that without nucleotides substitution rate, positive evolution pronounced in respiratory syndrome. Since transitions are more dominant than transversions, this indicates positive selection in a short span due to multiple substitutions. Consequently, it is essential to take account of transition/transversion rates to correctly confine the evolutionary information when unequal transitional ratios among respiratory syndrome sequences exist. SARS-CoV-2, SARS-CoV, and MERS-CoV simulation results demonstrate that lower to higher transition/transversion ratios favor the positive evolution due to synonymous substitution as recessive compared to nonsynonymous. The proximity-based on the p-distance between viral strains implies SARS-CoV-2 have substitution rate higher than SARS-CoV, while MERS-CoV stands in midway. Divergence time, dN, and dS output parameters are data mining from 4-fold degenerate sites at the 3-codon positions and noncoding sites. The consequences explain that dN-dS are inversely proportional to divergence time, and dN substitution favors positive evolution. The above discussion is based on the estimate selection for HyPhy and Z-test results for three different respiratory syndrome strains that also agree with positive evolution and the average distance between them.

Compared nucleotide substitution models, especially HKY and T-Nei, Fels1981, and GTR for nucleotide sequences and establish that dN/dS ratio goes beyond than one, tend towards positive selection. It has been concluded that in the lack of transition/transversion, synonymous and nonsynonymous substitution rates tending to positive evolution for entire the methods NG (JC), LWL, PBL, and Kumar. The comparative results obtained from consistent and continuous analysis signify that higher transition/transversion rates show lesser dN-dS under positive evolution and reject the null hypothesis. Moreover, it is also observed that substitution models and dN-dS having a high impact on divergence time. [Fig f1-gi-20058]

## Figures and Tables

**Table 1.** Simulative estimate of S, N, dS, dN, and normalized (dN-dS) average value using codon through HyPhy using ML approach of SARS-CoV-2, SARS-CoV, and MERS-CoV

Model	Syn sites (S)	Nonsyn sites (N)	dS	dN	dN-dS	Normalized dN-Ds
Fels1981	1,104.58	4,157.41	1,666.33	1,893.72	227.38	6.7254
HKY	1,037.75	4,510.25	1,363	2,100.25	737.25	1.1325
T-Nei	1,010.41	4,657.58	1,507.7	2,202.14	694.43	2.2069
GTR	1,010.41	4,657.58	1,507.7	2,202.14	694.43	2.2069

ML, maximum likelihood; SARS-CoV-2, severe acute respiratory syndrome coronavirus 2; SARS-CoV, severe acute respiratory syndrome coronavirus; MERS-CoV, Middle East respiratory syndrome coronavirus; Fels1981, Felsenstein 1981 model; HKY, Hasegawa-Kishino-Yano model; T-Nei, Tamura-Nei model; GTR, General Time Reversible model.

**Table 2.** Average estimations (dN-dS) of Z-codon‒based test selection for all sequence pairs

Evolutionary model	dN-dS		
	Mathematical correlation	dN = dS Neutral	dN > dS Positive
NG (P) $\left\{pS=\frac{S_{d}}{S},\;pN=\frac{N_{d}}{N}\right\}$	dN=-34ln*1-43*pN	‒1.397	‒1.419
	dS=-34ln*1-43*pS		
NG (JC) $\left\{pS=\frac{S_{d}}{S_{R}},\;pN=\frac{N_{d}}{N_{R}}\right\}$		0.034	0.043
LWL (K-2)	$dS=3\frac{\left[L_{2}A_{2}+L_{4}(A_{4}+B_{4})\right]}{\left(L_{2}+3L_{4}\right)}$	0.022	0.026
	$dN=3\frac{\left[L_{2}B_{2}+L_{0}(A_{0}+B_{0})\right]}{\left(2L_{2}+3L_{0}\right)}$		
PBL (K 2)	$dS=B_{4}+\frac{\left(L_{2}A_{2}+L_{4}A_{4}\right)}{\left(L_{2}+L_{4}\right)}$	0.012	0.019
	$dN=A_{0}+\frac{\left(L_{0}B_{0}+L_{2}B_{2}\right)}{\left(L_{0}+L_{2}\right)}$		
Kumar method (K-2)	$P_{0}=\frac{S_{0}+S_{2N}}{L_{0}+L_{2C}}$	0.811	0.831
	$P_{2}=\frac{S_{0}+S_{2S}}{L_{2S}+L_{2C}}$		

Number of synonymous differences (Sd) and nonsynonymous (Nd) differences. Normalized number of synonymous sites (S) and nonsynonymous sites (N), numbers of synonymous (*Sd*) to normalize using the number of potential synonymous sites (*S_R_*), L_0_, L_2_, and L_4_ are the number of 0-fold, 2-fold, and 4-fold degenerate sites, respectively. *L*_0_, *L*_2S_, *L*_2C_, and *L*_4_ are the numbers of 0-fold, simple 2-fold, complex 2-fold, and 4-fold degenerate sites. NG (P), Nei-Gojobori method (proportion); NG (JC), Nei-Gojobori method (Juke Cantor); LWL, Li-Wu-Luo method (K-2); PBL, Pamilo-Bianchi-Li method (K-2).
